# Neutralization of MERS coronavirus through a scalable nanoparticle vaccine

**DOI:** 10.1038/s41541-021-00365-w

**Published:** 2021-08-24

**Authors:** Mona O. Mohsen, Dominik Rothen, Ina Balke, Byron Martina, Vilija Zeltina, Varghese Inchakalody, Zahra Gharailoo, Gheyath Nasrallah, Said Dermime, Kaspars Tars, Monique Vogel, Andris Zeltins, Martin F. Bachmann

**Affiliations:** 1 Department of BioMedical Research, University of Bern, Bern, Switzerland; 2 Department of Immunology RIA, University Hospital Bern, Bern, Switzerland; 3 Translational Cancer Research Facility and Clinical Trials Unit, Interim Translational Research Institute, Hamad Medical Corporation, Doha, Qatar; 4 Latvian Biomedical Research & Study Centre, Ratsupites iela1, Riga, LV Latvia; 5 Erasmus Medical Center, department of Viroscience, Rotterdam, The Netherlands; 6 Artemis Bio-Support, Delft, The Netherlands; 7 Research Complex, Qatar University, Doha, Qatar; 8 Jenner Institute, Nuffield Department of Medicine, University of Oxford, Oxford, UK; 9 International Immunology Centrer, Anhui Agricultural University, Hefei, China

**Keywords:** Protein vaccines, Immunology

## Abstract

MERS-CoV continues to cause human outbreaks, so far in 27 countries worldwide following the first registered epidemic in Saudi Arabia in 2012. In this study, we produced a nanovaccine based on virus-like particles (VLPs). VLPs are safe vaccine platforms as they lack any replication-competent genetic material, and are used since many years against hepatitis B virus (HBV), hepatitis E virus (HEV) and human papilloma virus (HPV). In order to produce a vaccine that is readily scalable, we genetically fused the receptor-binding motif (RBM) of MERS-CoV spike protein into the surface of cucumber-mosaic virus VLPs. The employed CuMV_TT_-VLPs represent a new immunologically optimized vaccine platform incorporating a universal T cell epitope derived from tetanus toxin (TT). The resultant vaccine candidate (mCuMV_TT_-MERS) is a mosaic particle and consists of unmodified wild type monomers and genetically modified monomers displaying RBM, co-assembling within *E. coli* upon expression. mCuMV_TT_-MERS vaccine is self-adjuvanted with ssRNA, a TLR7/8 ligand which is spontaneously packaged during the bacterial expression process. The developed vaccine candidate induced high anti-RBD and anti-spike antibodies in a murine model, showing high binding avidity and an ability to completely neutralize MERS-CoV/EMC/2012 isolate, demonstrating the protective potential of the vaccine candidate for dromedaries and humans.

## Introduction

Over the past two decades, different highly pathogenic coronaviruses caused several major outbreaks as seen with severe acute respiratory syndrome (SARS) in 2002 in China, Middle East Respiratory Syndrome (MERS) in 2012 in Saudi Arabia^1^ and most recently with the severe outbreak of SARS-CoV-2 that started in Wuhan city of China in 2019^2^. Following the first registered MERS epidemic in 2012 in Saudi Arabia, MERS-CoV continues to cause outbreaks in animals and humans in >27 countries worldwide including Saudi Arabia, Qatar, Oman, United Arab Emirates, Bahrain, Egypt, Jordan, USA, UK as well as China.

The WHO MERS report, published on January 2020 has recorded a total number of 2519 confirmed cases including 866 associated deaths (case-fatality rate of 34.3%) worldwide. The majority of the cases were reported in Saudi Arabia. Death occurred in patients between 45–85 years with comorbidities and no changes in the demographic and epidemiological characteristics have been reported when compared to the periods in 2014–2020^3^. Interestingly, a recent case series study has reported that 12% of the total numbers of MERS-infected cases admitted to the intensive care unit (ICU) (from March 14, 2020 to October 19, 2020) showed coinfection with SARS-CoV-2^4^. This study raises new concerns of the possibility of interaction between both coronaviruses.

Similar to other coronaviruses, MERS-CoV is a zoonotic virus originating from bats, which probably serve as natural reservoirs (Lu et al., 2015), while dromedary camels most likely are intermediate hosts where the virus, however, remains endemic and causes mild symptoms similar to seasonal CoVs in humans (Abdulaziz N. Alagaili et al., 2015). Transmission of MERS-CoV among humans is considered rare but yet very possible. Due to the high mortality rate of MERS virus (∼25–35%), the WHO declared the need for effective countermeasures such as vaccines for dromedaries as well as for humans. Indeed, this human to human transmissible disease represents a major threat and should gain political and public health attention before a next major outbreak.

Coronaviruses infect host cells via their spike glycoprotein which consists of S1 and S2 subunits. The S1 of MERS-CoV is encoded by amino acids (a.a.) 376–606 and contains the receptor-binding domain (RBD) that is composed of a core subdomain and a receptor-binding motif (RBM; a.a. 484–567). The RBM is the main domain that interacts with the DPP4 receptor and facilitates viral entry into target cells^5,6^. After attaching to the cell surface, peptidases from the host cleave the S proteins, exposing the fusion peptide located in the S2 subunit. Such process leads to fusion of the viral and host cell membranes which enables the viral genome to enter the host cell. As for many other viruses, fusion mostly occurs in endosomes causing the release of the viral genome into the host cytosol. Finally, the viral RNA becomes available for translation and replication, resulting in assembly and packaging of new viral particles which are released from the cell^7^. Accordingly, the RBD and in particular the RBM domain may be considered the principal target against MERS-CoV for vaccine development.

Previous approaches for developing MERS-CoV vaccines included DNA vaccines, viral vectors, protein-based platforms or inactivated viruses^8^. The delivery of MERS-CoV S antigen by means of DNA vaccination^9^, viral vectors (modified vaccinia Ankara MVA or adenovirus)^10,11^, nanoparticles^12^ or RBD-based subunit vaccines^13^ have proved efficacy in preclinical models against MERS-CoV. Several vaccines are currently in phase I clinical trials. Despite the promising results and active research on this area, neither antiviral drugs nor vaccines have been approved for veterinary or medical use against MERS^14^.

Virus-like particles (VLPs) are nanoscale multiprotein particles that mimic real viruses. In comparison to inactivated or live-attenuated vaccine platforms, VLP-based vaccines are capable of inducing a robust humoral immune response without the risk of reversion to virulence^15^ and do not require BSL-3 facility. Due to their nano-size dimensions, they readily reach draining lymph nodes where they induce a strong immune response^16,17^. Such rapid draining kinetics improves the exposure of B cells to native VLPs and reduces potentially systemic inflammatory responses^18^. The traditional VLP platform has been used in several marketed vaccines against Hepatitis B Virus (HBV), Hepatitis E Virus (HEV), Human Papilloma Virus (HPV) as well as malaria^19^.

Cucumber mosaic virus-like particles (CuMV-VLPs) are derived from a plant virus. We have recently developed an immunologically optimized platform based on CuMV-VLPs, by incorporating a universal tetanus toxin (TT), a T helper (T_H_) cell epitope into the particle (CuMV_TT_-VLPs)^20^. This results in enhanced B cell / T_H_ cell interactions, especially important for elderly people and individuals with weak immune responses. This platform has reached several proofs of concepts in various animal species (reviewed in^20,21^). Furthermore, our newly developed vaccine platform is self-adjuvanted with prokaryotic ssRNA which is packaged within VLPs during the expression and assembly process in *E*. coli. The self-packaged adjuvant serves as TLR7/8 ligand and promotes the activation of antigen-presenting cells (APCs) and B cells^22–25^.

Several methods can be used to display antigen epitopes on the surface of VLPs; these methods include chemical coupling and genetic fusion techniques. In our previous studies we used chemical coupling strategies such as SMPH or Cu-free click chemistry cross-linkers^26–28^. In this study, we have chosen to genetically insert the viral antigen into the VLP protein, enabling future GMP-compatible production at very large scale at low cost. The size of the engineered mCuMV_TT_-MERS particles are in the nanoscale of ∼40–50 nm and densitometric analysis suggests an approximate 40% incorporation of RBM. On the surface of the VLPs, RBM exhibits the right conformation as demonstrated by binding of the viral receptor DPP4. Here we show that our new vaccine is immunogenic in murine models and induces high RBD and spike specific -antibody titers which are capable of blocking the replication of MERS-CoV/EMC/2012 isolate in vitro.

## Results

### Efficient incorporation of MERS receptor-binding motif (RBM) into mosaic CuMV_TT_ nanoparticles

We have used our immunologically optimized plant-derived cucumber-mosaic VLPs (CuMV_TT_-VLPs) to develop a mCuMV_TT_-MERS nanovaccine candidate. The icosahedral *T* = *3* CuMV_TT_-VLPs are identical to the parent VLPs where subunits A are arranged in pentamers and subunits B/C are arranged in hexamers^25^. CuMV_TT_-VLPs incorporate a tetanus toxin (TT) epitope which is a universal T cell epitope binding to essentially all HLA-DR molecules. The incorporated TT epitope is displayed at the interior surface of the particles, allowing the VLPs to self-assemble without altering their icosahedral geometry or interfering with epitopes to be displayed on the exterior surface^20,29^. Furthermore, displaying the TT epitope on the interior surface of the particle prevents interference with TT-specific antibodies. TT-epitope incorporation is believed to improve the immune response specifically in aged people due to enhanced interaction between TT-specific T_H_ cells and epiotpe specific B cells, and is based on the fact that pre-existing immunity to TT epitope is broad in humans due to extensive vaccination programs. We have incorporated the coding sequencine of the receptor-binding motif (RBM; a.a. 484–566) of MERS-CoV protein into CuMV_TT_ nanoparticles. The RBM domain has been selected based on previous studies on SARS-CoV-2, where RBM proved to be a potent vaccine epitope^30,31^.

**Fig. 1:**
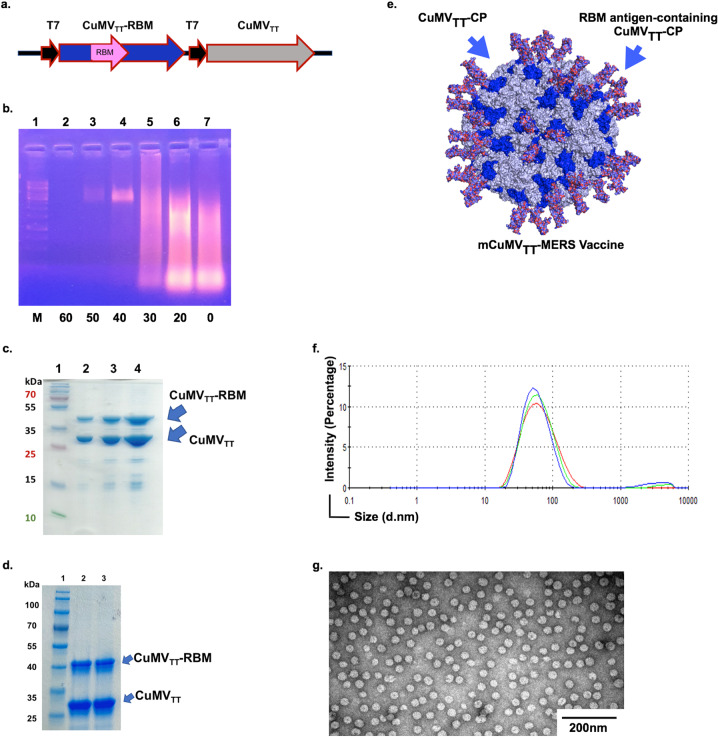
Efficient incorporation of MERS antigen receptor-binding motif (RBM) into mosaic CuMV_TT_-VLPs. **a** Gene map in pETDuet-1 for expression of a mosaic mCuMV_TT_-MERS vaccine candidate based on self-assembling of unmodified (CuMV_TT_) and genetically modified monomers displaying RBM (CuMV_TT_-RBM). **b** Agarose gel analysis of sucrose gradient fractions, Lane 1: molecular marker, Lanes 2–7: 60 – 0 sucrose gradient fractions of mCuMV_TT_-MERS. Fractions 40% and 50% contain VLPs, incorporated host cell ssRNA is stained with ethidium bromide. **c** SDS-PAGE stained with coomassie blue stain, Lane 1: protein marker, Lanes 2–4: mCuMV_TT_-MERS vaccine showing a band of unmodified CuMV_TT_ monomer of ~ 28 kDa and RBM antigen-containing CuMV_TT_ monomer of ~ 42 kDa (2, 4, and 8 µg of protein loaded). **d** SDS-PAGE stained with coomassie blue stain showing the stability of mCuMV_TT_-MERS vaccine after 1 month in RT, Lane 1: protein marker, Lane 2: mCuMV_TT_-MERS vaccine. **e** 3D model of mCuMV_TT_-MERS vaccine illustrating unmodified CuMV_TT_ monomer (in gray color) and the genetically modified CuMV_TT_ monomer (in blue color) displaying RBM (in pink color) and a video (Supplementary Data). **f** Dynamic light scattering. **g** Electron microscopy (EM) of mCuMV_TT_-MERS vaccine, scale 200 nm.

The vaccine candidate was engineered as a mosaic particle where unmodified wild-type monomers and genetically modified monomers (incorporating RBM) assemble together forming a VLP (Fig. 1a). Purification of mCuMV_TT_-MERS was carried out using sucrose gradient (Fig. 1b). The unmofidied monomer has a size of ~28 kD while the ones incorporating RBM have an apparent size of ~42 kDA as shown in SDS-PAGE (Fig. 1c). Furthermore, the production of mCuMV_TT_-MERS in *E*. coli system allowed spontaneous packaging of ssRNA which serve as toll-like receptor (TLR) 7/8 agonist. We have also tested the stability of the vaccine candidate and the results indicate that the platform is thermostable at 4 °C for more than a year and for about a month at RT (Fig. 1d). The size of the engineered mCuMV_TT_-MERS particles is ~40–50 nm. Densitometric analysis suggests an approximate 40% incorporation of RBM. As each VLP contains 180 capsid protein (CP) subunits, 40% results in ~70 RBM antigen-containing CuMV_TT_-CP molecules per 1 VLP (Fig. 1e and Video 1). Dynamic light scattering revealed a homogenous and uniform peak of hydrodynamic radius (R_H_) of ~ 94 nm (Fig. 1f); the observed peak is larger than the expected size due to water molecules surrounding the VLPs. Electron microscopy imaging confirms the assembly of icosahedral particles and indicated no signs of VLP aggregation following expression in *E*. coli (Fig. 1g).

### RBM displayed on the surface of mCuMV_TT_-MERS vaccine binds the human receptor DPP4

**Fig. 2:**
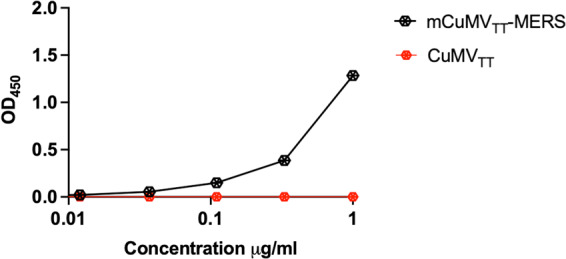
RBM displayed on the surface of mCuMV_TT_-MERS vaccine binds human receptor DPP4. DPP4 binding of mCuMV_TT_-MERS vaccine candidate and CuMV_TT_ as a control. Plates coated with 1 μg/ml of DPP4. Binding revealed with an anti-CuMV mAb. One representative of three similar experiments is shown.

To confirm the antigenicity of mCuMV_TT_-MERS as well as the correct folding and confirmation of RBM displayed on the particle’s surface, we performed a receptor binding assay. To this end, the human receptor DPP4 was coated on ELISA plate. mCuMV_TT_-MERS vaccine was added, in parallel to CuMV_TT_ as a control. Anti-CuMV_TT_ antibodies were used as a secondery antibody to detect receptor bound VLPs. The results revealed that mCuMV_TT_-MERS can bind to DPP4 receptor indicating correct folding of RBM on the surface of the VLP while the control did not show any binding (Fig. 2).

### Vaccination with mCuMV_TT_-MERS elicits a strong humoral immune response

**Fig. 3:**
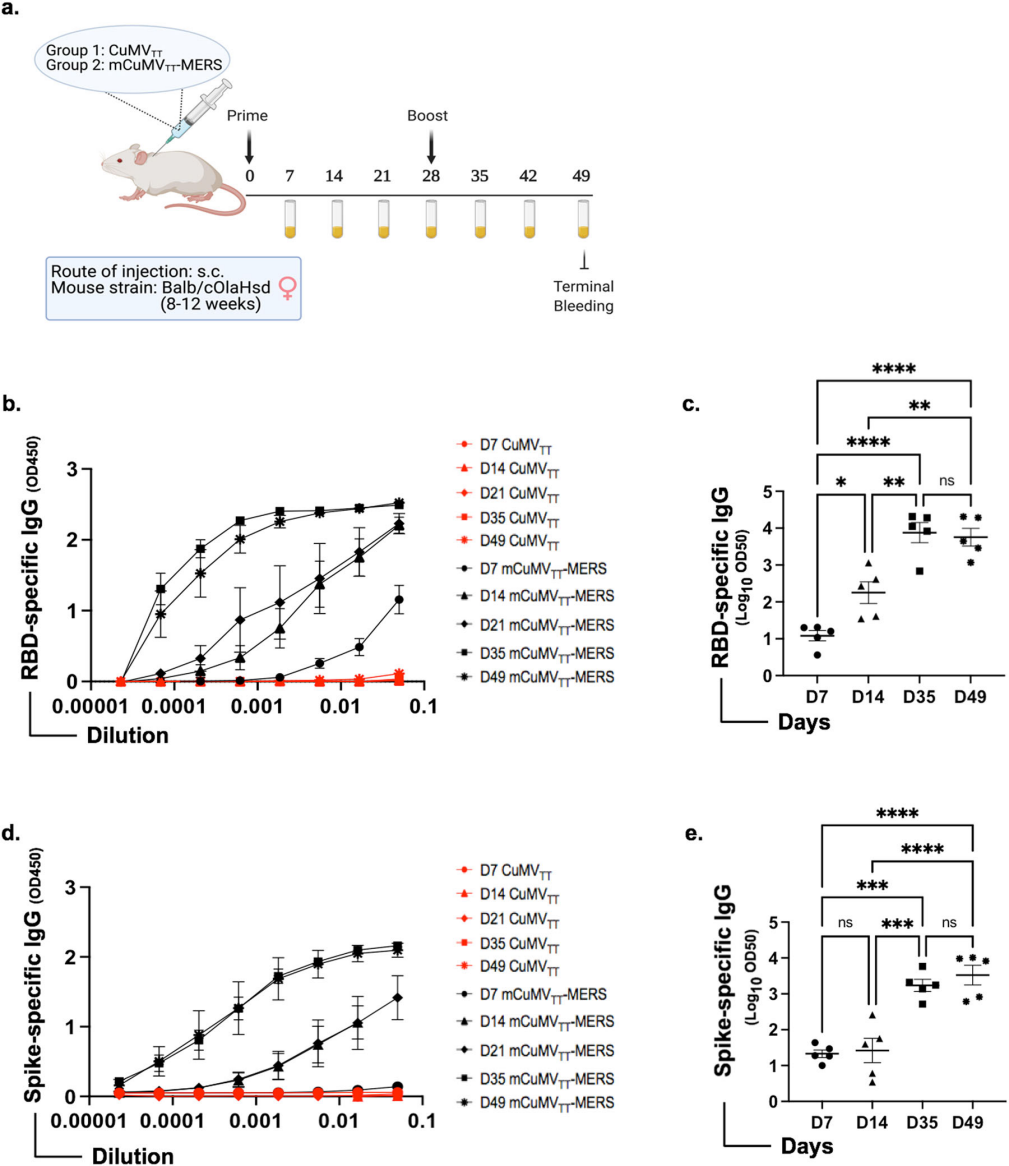
Vaccination with mCuMV_TT_-MERS elicits a strong humoral immune response. **a** Overview of vaccination regimen (prime on day 0 and boost on day 28), bleeding time points and groups. Serum samples were collected from mice on a weekly basis. Figure created with BioRender.com. **b** RBD-specific IgG titer for the groups vaccinated with CuMV_TT_ control or mCuMV_TT_-MERS on days 7, 14, 21, 35, and 49 measured with OD_450nm_. **c** Log_10_ OD_50_ values (mean ± SEM) of RBD-specific IgG titer for the groups vaccinated with mCuMV_TT_-MERS on days 7,14, 35, and 49 (data from B). **d** Spike-specific IgG titer for the groups vaccinated with CuMV_TT_ or mCuMV_TT_-MERS on days 7,14,21,35, and 49 measured with OD_450_. **e** Log_10_ OD_50_ values (mean ± SEM) of spike-specific IgG titer for the groups vaccinated with mCuMV_TT_-MERS on days 7,14, 35, and 49 (data from D). Statistical analysis using one-way ANOVA. Control group *n* = *5* and vaccine group *n* = *5*. One representative of 2 similar experiments is shown. The value of *p* < 0.05 was considered statistically significant (*p < 0.01, **p < 0.001, ***p < 0.0001).

The immunogenicity and the induction of a humoral immune response in murine models upon vaccination with mCuMV_TT_-MERS was examined by ELISA. BALB/c mice were primed s.c. with 100 μg CuMV_TT_ as a control or with mCuMV_TT_-MERS vaccine candidate without any adjuvant. A booster dose was given 28 days later. Mice were bled on a weekly basis and terminally bled on day 49 as illustrated in Fig. 3a. Total IgG was measured against RBD as well as against spike protein of MERS-CoV. RBD-specific IgG antibodies were detected as early as seven days post priming in the group vaccinated with mCuMV_TT_-MERS. The induced response against RBD increased ∼10-fold following priming and reached the peak one week after the booster dose (Fig. 3b, c). The response against spike protein was somewhat delayed compared to the response against RBD protein. Nevertheless, an ~100-fold increase in IgG titer was detected three weeks after priming on day 21 and further increased following the booster dose (Fig. 3d, e). No RBD or spike specific antibodies were detected prior to vaccination.

### mCuMV_TT_-MERS vaccine induces RBD and spike-specific IgG subclasses as well as isotype switching to IgA

**Fig. 4:**
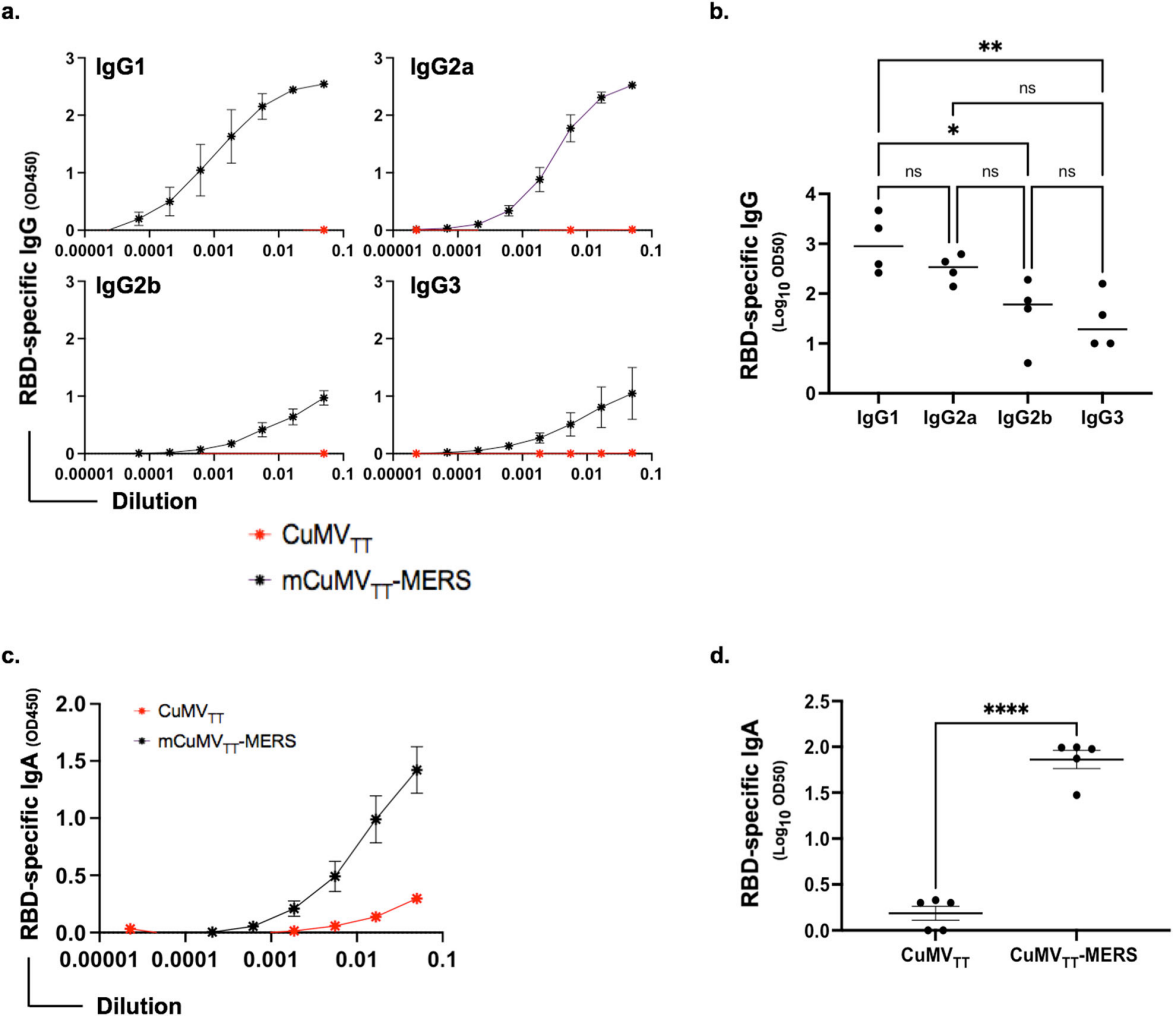
mCuMV_TT_-MERS vaccine induces RBD and spike-specific IgG subclasses as well as isotype switching to IgA. **a** RBD-specific IgG1, IgG2a, IgG2b and IgG3 titers for the groups vaccinated with CuMV_TT_ control or mCuMV_TT_-MERS measured with OD_450_. **b** Log_10_ OD_50_ values (mean ± SEM) of RBD-specific IgG1, IgG2a, IgG2b and IgG3 titer for the groups vaccinated with mCuMV_TT_-MERS, (*p* value 0.0202 for IgG1 vs IgG2b, *p* value 0.0096 for IgG1 vs IgG3). Statistical analysis using One-way ANOVA. Control group *n* = *4* and vaccine group *n* = *4*. **c** RBD-specific IgA titer for the groups vaccinated with CuMV_TT_ control or mCuMV_TT_-MERS measured with OD_450_. **d** Log_10_ OD_50_ values (mean ± SEM) of RBD-specific IgA titer for both groups, (*p* value < 0.0001). Statistical analysis using Student’s *t* test. Control group *n* = *5* and vaccine group *n* = *5*. One representative of two similar experiments is shown. The value of *p* < 0.05 was considered statistically significant (*p < 0.01, **p < 0.001, ***p < 0.0001).

We next evaluated the ability of the engineered vaccine candidate to induce IgG subclasses and IgA using the sera collected at day 49. To this end, ELISA plates were coated with RBD or spike proteins of MERS-CoV. The results showed that mCuMV_TT_-MERS vaccination induces the production of all RBD-specific IgG subclasses (IgG1, IgG2a, IgG2b and IgG3) dominated by IgG1 and IgG2a (Fig. 4a, b). IgG subclasses could also be detected against the spike protein as shown in Supplementary Fig. 1. Isotype switching to RBD-specific IgA antibodies was also induced following vaccination with mCuMV_TT_-MERS (Fig. 4c) and reached an OD_50_ of greater than 1:100 (Fig. 4d).

### Induction of high avidity antibodies after vaccination with mCuMV_TT_-MERS

**Fig. 5:**
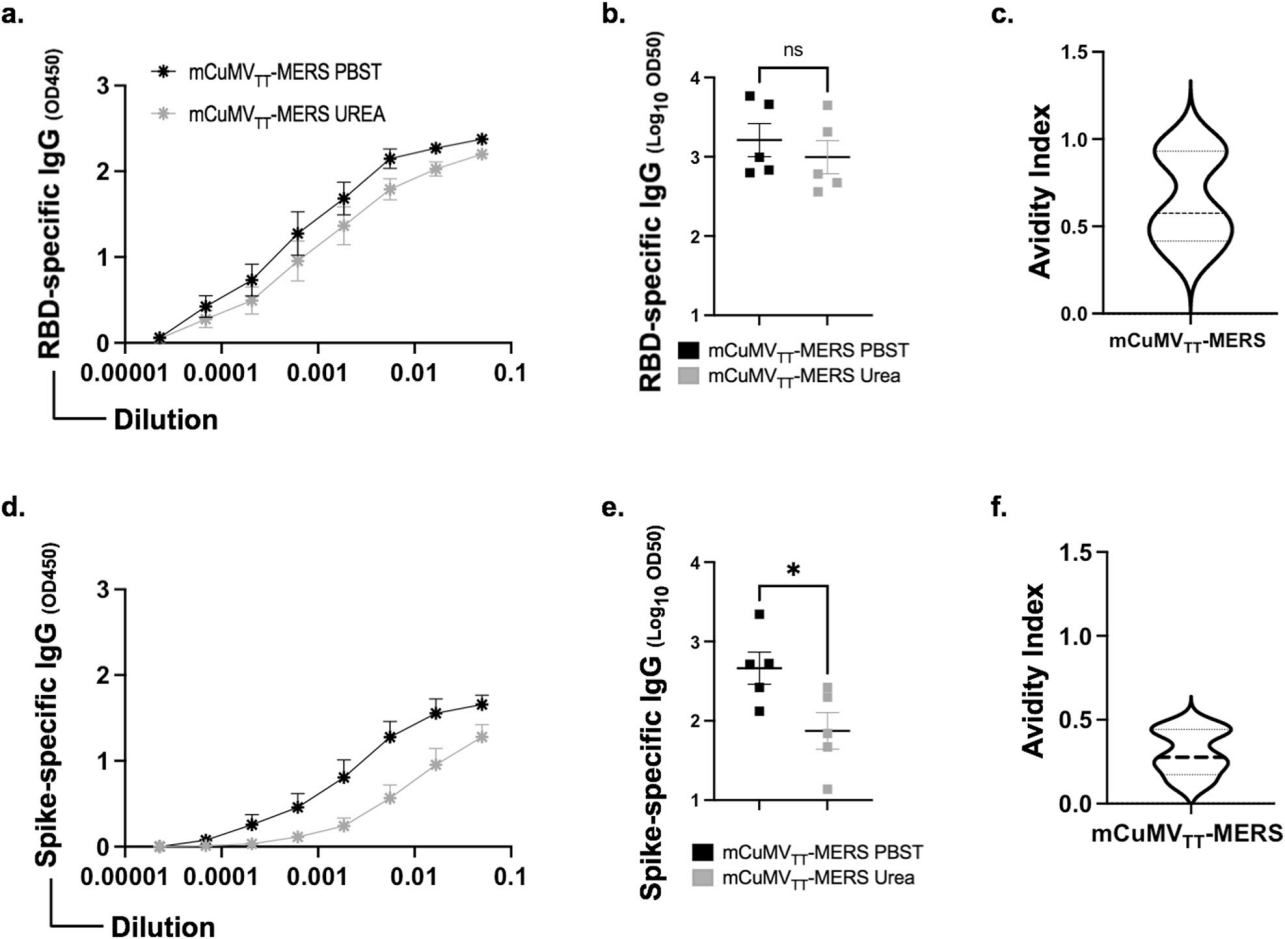
Induction of high avidity antibodies after vaccination with mCuMV_TT_-MERS. **a**, **d** RBD- and spike specific IgG titer for the group vaccinated with mCuMV_TT_-MERS vaccine on day 35 measured with OD_450_. After serum incubation one plate was treated with PBS+0.05% Tween 20 and the other plate with 7 M Urea in PBS+0.05% Tween 20. **b**, **e** Log_10_ OD_50_ values (mean ± SEM) of RBD- and spike-specific IgG titers (shown in **a** and **d**) for the group vaccinated with mCuMV_TT_-MERS, (*p* value 0.4860 in **b** and 0.0327 in **e**). **c**, **f** Avidity index showing the percentage of high avidity RBD- and spike-specific IgG antibodies calculated with the data from (**a**) or (**d**). Statistical analysis using Student’s *t* test. Control group *n* = *5* and vaccine group *n* = *5*. One representative of 2 similar experiments is shown. The value of *p* < 0.05 was considered statistically significant (*p < 0.01, **p < 0.001, ***p < 0.0001).

To further characterize the induced RBD-specific IgG antibodies, we performed avidity ELISAs using sera collected one week following the booster dose. The results showed that the mCMV_TT_-MERS vaccine is capable of generating high avidity antibodies (Fig. 5a). Essentially no difference was detected between the ELISA plates treated with PBST or 7 M UREA (to eliminate low avidity antibodies) for the group vaccinated with mCuMV_TT_-MERS (Fig. 5b). We also calculated the avidity index by comparing the two treated plates for the group vaccinated with mCuMV_TT_-MERS. The mean index was ~0.6, indicating that around 60% of the total IgG antibodies are of high avidit for RBD (Fig. 5c). The avidity of the spike-specific antibodies was also tested (Fig. 5d). The small difference (*p*. 0.0327) detected between the sera treated with PBST or Urea indicate that about 30% of the generated antibodies could recognize the spike protein with high avidity (Fig. 5e, f).

### mCuMV_TT_-MERS-induced antibodies block binding of RBD to the viral receptor DPP4 and neutralize the wild-type MERS virus

**Fig. 6:**
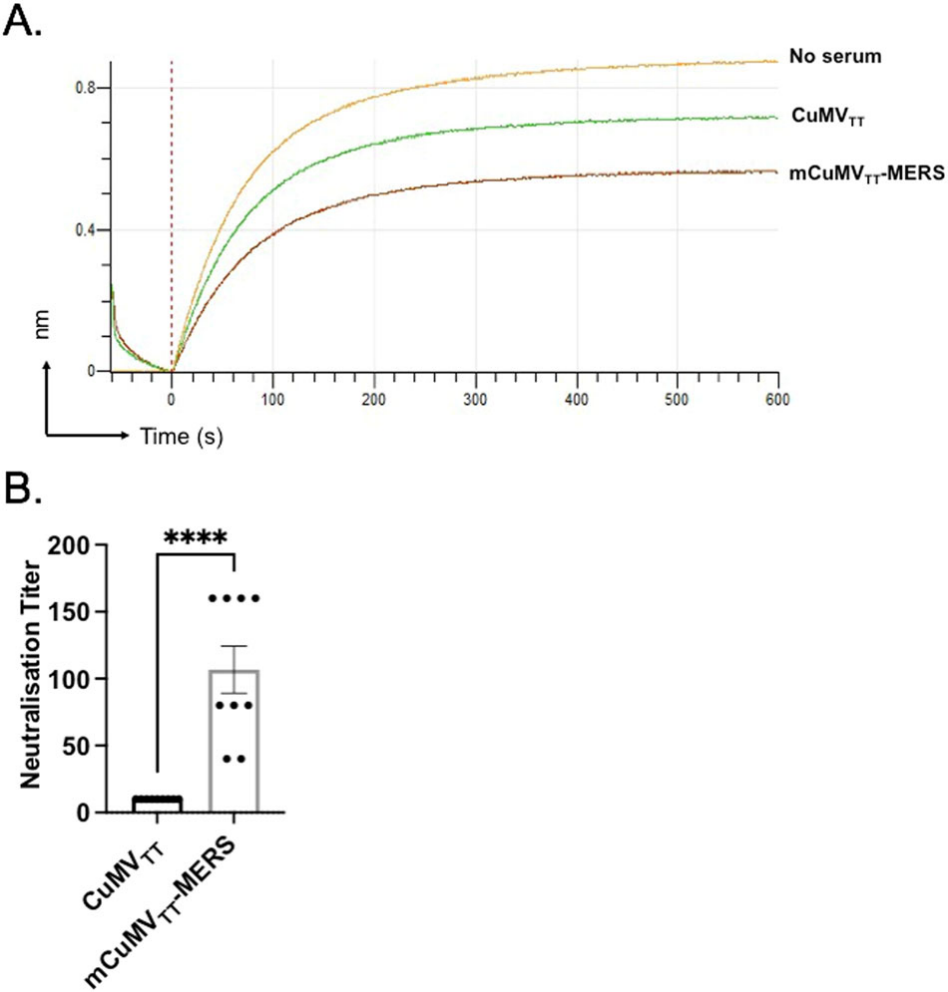
mCuMV_TT_-MERS blocks binding to RBD to the viral receptor DPP4 and neutralizes wild-type MERS virus. **a** BLI-evaluation of DPP4 binding to RBD in the presence of vaccinated mice sera. Binding of DPP4 to BLI biosensors coated with RBD-his tag is significantly reduced in the presence of mouse sera (1:10 dilution, d35) after 2nd vaccination with mCuMV_TT_-MERS (brown line) than with CuMV_TT_ control (green line) or with no serum (line orange) consistent with DPP4-blocking activity. **b** Neutralization titers (CPE) of the groups vaccinated with CuMV_TT_ control and mCuMV_TT_-MERS, *p* < 0.0001. Statistical analysis using Student’s *t* test. Control group *n* = *10* and vaccine group *n* = *9*. One representative of two similar experiments is shown. The value of *p* < 0.05 was considered statistically significant (*p < 0.01, **p < 0.001, ***p < 0.0001).

To determine whether the induced IgG antibodies can compete with the human receptor DPP4 for binding to RBD, we performed antibody competition assays using biolayer interferometry. RBD-His was immobilized onto anti-His biosensors and competitions of DPP4 to RBD was quantified as reduction in RBD binding in the presence of serum samples. As shown in Fig. 6a, sera of mice immunized with mCuMV_TT_-MERS were able to inhibit DPP4 binding to RBD. For comparison, sera from control mice immunized with CuMV_TT_ alone were used. The results indicate that immunization with mCuMV_TT_-MERS induced anti-RBM antibodies capable of inhibiting the binding of MERS-RBD to DPP4.

To determine the neutralizing capacity of the induced antibodies, the sera were tested for their ability to prevent cytopathic effects of MERS-CoV/EMC/2012 on Vero cells in vitro (CPE-based neutralization assay). The titer is indicated as the dilution at which an inhibiting effect was visible and the cytopathic effect was fully prevented. The group vaccinated with mCuMV_TT_-MERS fully inhibited the cytopathic effect of the virus while the control group CuMV_TT_ did not show any inhibition, demonstrating a strong neutralizing capacity of the induced antibodies (Fig. 6b).

## Discussion

Few MERS-CoV vaccines based on VLPs have been reported so far in preclinical studies. Wang et al. have developed a chimeric VLP vaccine expressing RBD of MERS-CoV utilizing canine parvovirus (VP2). The vaccine could induce a good response in murine models when delivered with Poly (I:C)^12^. The same group has tested another VLP vaccine containing MERS-CoV S, E and M proteins in rhesus macaques which also showed efficacy^32^.

In the present study, we have engineered a vaccine candidate against MERS-CoV by genetically fusing the RBM domain of MERS-CoV into our immunologically optimized VLP-platform derived from the cucumber-mosaic virus (CuMV_TT_-VLPs). Designing the vaccine-candidate in this study as a mosaic vaccine was an essential step to allow the formation of icosahedral *T* = 3 VLPs^25^. Each engineered VLP incorporates ~70 RBM fused to the CP (40%). Expression in *E*. coli facilitateted the packaging of bacterial ssRNA which serves as TLR7/8 ligand. Previous studies demonstrated that VLP-based vaccines are capable of inducing IL-21-independent secondery plasma cells only in the presence of TLR7/8 agonists (ssRNA). Consequently, high levels of IgG antibodies with high affinity are produced within few days after challenge^22,33^. ssRNA packaged in VLPs polarizes the induced immune response towards T_H_1 rather than T_H_2, which prevents enhanced disease after viral challenge at least in preclinical models^2^. In addition, we incorporated a tetanus toxin (TT) epitope in the interior surface of our CuMV_TT_-VLPs. This may not necessarily affect the antibody response in mice or dromedaries as they are not pre-immunized against TT. However, the immunogenicity will be enhanced in humans due to previous tetanus vaccinations, likely helpful in elderly people and immunodeficient individuals who are known to require more immunogenic vaccines as compared to the general population. We have chosen the RBM-MERS as our vaccine target based on several grounds: (1) we found that genetically fusing the whole RBD domain of SARS-CoV-2 into VLPs hinders the assembly process of VLPs; on the other hand fusing RBM in AP205-VLPs resulted in an effective vaccine capable of neutralizing SARS-CoV-2^31^. (2) Based on previous studies for SARS-CoV-1 and 2, RBD is glycosyated and methylated. Such posttranslational modifications may be difficult to reproduce in bacteria and may result in the induction of low affinity, poorly protective antibodies. Importantly, RBM is neither glycosylated nor methylated and thus can directly interact with the human receptor MERS-DPP4. RBM is also the major target of neutralizing antibodies^34,35^ and therefore represents and ideal vaccine target. To our knowledge, we present here the first VLP-based vaccine displaying the RBM domain of MERS-CoV that can easily be produced in large amounts as it self-assembles in *E*. coli without the need of any further modifications.

Vaccine candidates should display antigens in an authentic native configuration in order to induce an effective B cell response^18^. Using Sandwich ELISAs, we have shown that mCuMV_TT_-MERS particles are able to detect and bind to the viral receptor DPP4. This confirms that the MERS RBM displayed and fused to the VLP, has the correct conformation which is important for the induction of the appropriate neutralizing antibody response.

We have not directly measured the induction of T_H_ cells responses by our vaccine candidate, mostly because the expected effector mechanism is induction of neutralizing antibodies. In addition, the size of the RBM-domain may be too small to reliably induce a T_H_ cell response in inbred mice. However, we have previously shown that VLP-specific T_H_ cell responses mediate isotype-switch for B cells specific for antigens displayed on the VLPs. Furthermore, the bacterial RNA packaged in VLPs, such as CuMV_TT_, drive CD8^+^ and T_H_1 responses.(44–46).

Mouse IgG antibodies vary in their immunological, biochemical as well as physiological properties. We have previously shown that TLR7/8 triggering in B cells induces subclass switch to IgG2a and IgG2b; bacterial RNA was most efficient at triggering such responses^23,36^. Vaccination with mCuMV_TT_-MERS enhanced the induction of all IgG subclasses which was dominated by IgG1 and IgG2a, other subclasses could also be detected to a much lesser extent. In addition, RNA loaded VLPs may also induce IgA responses, again in a TLR7/8 dependent manner^22,24^. This appears particularly important for MERS-CoV and other respiratory diseases-causing viruses, such as SARS-CoV-1 and −2, as IgA may be able to neutralize the virus locally in the lung without causing inflammation, a feature that may be particularly critical in patients with high viral load. IgA is the predominant Ig isotype in the mucosal tissue and has a very important role in the defense against respiratory infections^37^. Thus, an effective vaccine against respiratory viruses should induce IgA antibodies. It is therefore of key importance that our newly developed vaccine is able to induce a significant increase in serum IgA levels. Whether the increased serum IgA levels in mice can translate to correspondingly high IgA levels in humans and the mucosa needs to be confirmed.

Testing the quality of the RBD-specific antibodies has shown high avidity binding which is a critical parameter for an effective vaccine. UREA is interfering with the antibody-antigen binding by breaking up the hydrophobic bonds. We found that ~60% of the RBD specific IgG antibodies are of high avidity and bind strongly to its antigen even in the presence of UREA. Thus, RBM displayed on CuMV_TT_ induces high avidity antibodies.

The main goal for any anti-viral vaccine is the elicitation of neutralizing antibodies which are able to directly inhibit cellular infection and provide protection from disease^38^. For MERS-CoV, most neutralizing antibodies bind to the spike glycoprotein and inhibit viral attachement to the human receptor DPP4, hence blocking viral entry and intracellular replication. Our test sera were probed for their ability to inhibit the cytopathic effect of MERS-CoV/EMC/2012 isolate on Vero cells. Indeed, sera from vaccinated mice were able to fully neutralize viral replication, showing that the vaccine is able to induce protective antibodies which can shield the host’s cells from MERS-CoV infection.

Collectively, we have shown in this study that an engineered VLP-based vaccine can efficiently induce high specific anti-RBD and spike antibodies that effectively neutalize MERS-CoV. As MERS represents an immanent global threat to human health, it seems rational to further develop this vaccine candidate and make it availalbe for emergency use.

## Methods

### mCuMV_TT_-MERS vaccine candidate production, purification and analysis

*E. coli* C2566 cells were transformed with the pETDu-CMVB3d-MERS-M-CMV_TT_ plasmid containing RBM of MERS and the Ampicillin (Amp) resistance gene. After transformation, five individual colonies were inoculated in 4 ml 2TY (1.6% Trypton, 1.0% Yeast extract, 0.5% NaCl) with Amp (100 mg/l) and 0.1% glucose and cultured overnight at 37 °C without shaking. The next day, 1 ml of the starting culture was added to 20 ml 2TY, cultivated at 30 °C until an OD_600nm_ of 0.8 was reached and then induced with 0.2 mM IPTG and 5 mM MgCl_2_ and cultivated ON at 20 °C and 7 xg. After 18 h an OD_600n_m of 5.20 was reached and samples for SDS-PAGE were prepared. After SDS-PAGE analysis of individual clones the 5 20 ml cultures were pooled together (100 ml in total) and the biomass was collected by centrifugation. The pellet was frozen at −20 °C. To disrupt the cells, 10 ml of buffer (20 mM Tris, 5 mM EDTA, 5 mM Et-SH, 5% glycerol, 10% sucrose, pH 8.0) was added to resuspend and further treat the biomass with ultrasound (Hielscher 200, power 70%, pulse 50%, 16 min) on ice. Then, 0.5% TX-100 was added and the solution was rotated at 10 rpm overnight (ON) at 4 °C without centrifugation. The solution was then centrifugated for 10 min at 15,557 xg (Eppendorf 5804) and the pellet was discarded. The soluble fraction was loaded on the top of the sucrose gradient (20–60%; in buffer containing 20 mM Tris, 2 mM EDTA, 5% glycerol, 0.5% TX-100, pH 8.0) and centrifugated in Beckman SW32 rotor for 6 h at 106,559 xg at 18 °C. The gradient fractions (6 ml) were then removed from the bottom of the 38 ml tube. The mCuMV_TT_-MERS containing fraction (40 and 50% sucrose, pooled) was diluted 1:1 with buffer (20 mM Tris, 2 mM EDTA, 5% glycerol, pH 8.0). The VLPs were sedimented using Type 70 rotor (Beckman, 183,960 xg, 4 h, 4 °C). Then the pellet was dissolved ON in 2 ml of 20 mM Tris, 2 mM EDTA at 4 °C. The solution was clarified by centrifugation (5 min, 16,873 xg), the clarified solution overlaid on top of the 30% sucrose “cushion” solution in 20 mM Tris, 2 mM EDTA, 0.5% TX-100, pH 8.0 The VLPs were sedimented using Beckman TLA100.3 rotor (220,050 xg, 60 min, 4 °C). The pellet was solubilized in 1 ml of 20 mM Tris, 2 mM EDTA and clarified again by centrifugation (5 min, 16,873 xg). Obtained VLPs were characterized using SDS-PAGE, agarose gel, electron microscopy and dynamic light scattering. Protein concentration was determined using BCA test.

### Binding ELISA assay

To test if the vaccine can bind the relevant human receptor DPP4, the plates were coated with 1 µg/ml of DPP4 in PBS at a volume of 50 μl/well. The plate was incubated at 4 °C overnight. The plate was washed with PBS+Tween 0.01%. 50 μl/well of Superblock solution (Thermo Fisher, 37518) was added and the plate was incubated for 1 h at RT on a shaker. The blocking solution was flicked off and 50 μl of mCuMV_TT_-MERS or CuMV_TT_ (control) from 1 µg/ml was added to the first row of the plate followed by 1:3 dilution. The plate was incubated for 1 h at RT then washed with PBS+Tween 0.01%. Next, 50 μl of mouse anti-CuMV_TT_ monoclonal antibody (clone 1-1A8/ batch 2) at a concentration of 1 μg/ml was added to each well as a secondary antibody and incubated for 1 h at RT on a shaker. The plate was washed and 50 μl of the detection antibody; HRP labeled goat anti-mouse IgG Fc gamma at a dilution of 1:1000 in PBS-Casein 0.15% was added to each well. The plate was incubated for 1 h at RT. The plate was developed and OD450 reading was performed (BioTek, USA).

### Mice

Wild type Balb/cOlaHsd mice were purchased from Harlan. All in vivo experiments were performed using 8–12 weeks old female mice.

### Ethical approval

All animal procedures were conducted in accordance with the Swiss Animal Act (455.109.1 – September 2008, 5th) using License No. BE70/2018.

### Vaccination regimen

Wild type Balb/cOlaHsd mice (8–12 weeks, Harlan) were vaccinated subcutaneously (s.c.) in the neck with 100 μg with either mCuMV_TT_-MERS vaccine or CuMV_TT_ as a control in a volume of 100 μl without any adjuvants. The mice received a boost with an equal dose at day 28 and were terminally bled at day 56. Serum was collected on a weekly basis vial tail bleeding and the serum was isolated using Microtainer Tube (BD Biosciences, USA).

### ELISA (Enzyme-Linked Immunosorbent Assay)

To determine the total IgG antibody titers, ELISA Corning^TM^ 96-Well Half-Area Plates (Fisher Scientific, USA) were coated with 1 μg/ml MERS-CoV RBD, (a.a. 367–606) (Sino Biological, USA) or MERS-CoV Spike/S1 protein S1 Subunit, (a.a. 1–725), diluted in PBS in a volume of 50 µl/well. The plates were incubated overnight at 4 °C on a shaker. The next day the plates were washed with the ELISA washer (BioTek, 405 TS) with PBS-tween 0.01%. The plates were blocked using 100 μl/well PBS-Casein 0.15% and incubated at RT for 2 h on a shaker. The blocking solution was removed by flicking the plates. Serum samples were diluted 1:20 followed by 1:3 serial dilution except for the last row which was used as a negative control. The plates were incubated for 2 h at RT on the shaker. Plates were washed with PBS-tween 0.01% and secondary anti mouse IgG Fc gamma conjugated with Horse-Radish Peroxidase (Jackson Immunoresearch Cat. Nr. C840T69) (1:1000) was added 50 μl/well. The plates were incubated for another 1 h on a shaker, then washed and developed and OD450 reading was performed (BioTek, USA). Log_10_ OD_50_ values were calculated using GraphPad PRISM.

To assess the subclass antibody response, the same procedure was performed, except using a different secondary antibody equivalent to each subclass: Rat anti-mouse IgG1 (BD Pharmingen, Cat. 559628, 1:2000 dilution), biotinylated mouse anti-mouse IgG2a (Clone 8.3, BD Biosciences, USA, 1:1000 dilution), goat anti-mouse IgG2b (Invitrogen, Ref. M32407, 1:2000 dilution) and goat anti-mouse IgG3 (Southern BioTech, Cat No 1101-05, 1:4000 dilution).

To detect IgA antibodies the plates were coated with 1 μg/ml MERS-CoV RBD, (a.a. 367–606) (Sino Biological, USA) and goat anti-mouse IgA POX (ICN 55549, ID 91, 1:1000 dilution) was used as a secondary antibody. An additional step prior to serum incubation in order to deplete IgG was performed. 10 μl of Protein G beads (Invitrogen, USA) were transferred into a tube and placed into a magnet. The liquid was removed and 75.6 µl diluted sera in PBS-Casein 0.15% was added to the beads and mixed. The tube was incubated on a rotator at RT for 10 min. The tubes were placed back into the magnet and ELISA was carried out as described above.

### Avidity ELISA

To determine the avidity of IgG antibodies, two sets of plates were prepared. Both were coated with 1 µg/ml MERS-CoV RBD, (a.a. 367–606) (Sino Biological, USA). After serum incubation, one set of plates was washed three times for 5 min with 50 µl/well 7 M Urea in PBS+0.05% Tween 20 whereas the other set was washed with the same amount of PBS+0.05% Tween. In-between the washing steps, all the plates were washed with PBS+0.01% Tween 20 with the ELISA washer. The rest of the procedure is identical as described above.

### Competition assay (OCTET)

The antibody competitive binding activities of the induced antibodies was performed on an OCTET RED96e (Fortébio) instrument which allows real-time analysis due to the shift in the wavelength of the reflected light. Anti-Penta-HIS (HIS1K, Lot 2006292, FortéBio) biosensors were first loaded into biosensor microplates and pre-hydrated in BLI assay buffer (PBS, 0.1% BSA, 0.02% Tween 20) for 10 min. 96-well microplates were loaded with 200 ml per well. The assay plate was then prepared as follows: column 1 (BLI assay buffer), column 2 (15 μg/ml MERS-CoV spike protein fragment RBD, aa267–606, diluted in 200 μl BLI assay buffer) (Sino Biological, USA), column 3 (BLI assay buffer), column 4 (pooled sera from 5 mice diluted 1:10 in BLI assay buffer, samples from control mice vaccinated with CuMV_TT_-VLPs alone and mice vaccinated with mCuMV_TT_-MERS were used, two additional biosensors were used as control, one for baseline and one without serum sample), column 5 (BLI assay buffer), column 6 (50 nM of human receptor DPP4 (Sino Biological, USA) diluted in BLI assay buffer), column 11 (Regeneration buffer, 0.1 M glycine, pH 1.5) and column 12 (neutralization buffer, BLI assay buffer).

### Neutralization assay (cytopathic effect-based neutralization assay)

Serum samples from day 56 were heat-inactivated for 30 min at 56 °C. Subsequently, serum samples were diluted two-fold, started at 1:20 to 1:160. Then 100 TCID50 of MERS-CoV/EMC/2012 was added to each well and incubated at 37 °C for 1 h. Following incubation, the mixtures were added on a monolayer of Vero cells and incubated at 37 °C for an additional four days. After four days wells were inspected for presence of cytopathic effect (CPE). Titer is expressed as the highest serum dilution that fully inhibits formation of CPE.

### Statistical analysis

Data were analyzed and presented as (mean ± SEM) using Student’s *t* test or One-way ANOVA as mentioned in the figure legends, with GraphPad PRISM 9. The value of *p* < 0.05 was considered statistically significant (*p < 0.01, **p < 0.001, ***p < 0.0001).

### Reporting summary

Further information on research design is available in the Nature Research Reporting Summary linked to this article.

## Supplementary information

Supp Material - Vaccination with mCuMVTT-MERS elicits a strong humoral immune response.

REPORTING SUMMARY

MERS-movie7

## Data Availability

The datasets generated during and/or analyzed during the current study are available from the corresponding author on reasonable request.
