# Supplementary material for: Neutralization of MERS coronavirus through a scalable nanoparticle vaccine
Source: NPJ Vaccines. 2021 Aug 24;6:107. doi: 10.1038/s41541-021-00365-w (PMC8384877; doi:10.1038/s41541-021-00365-w)
Supplement: Supplementary file 1 — Supp Material - Vaccination with mCuMVTT-MERS elicits a strong humoral immune response. [file 41541_2021_365_MOESM1_ESM.pdf]

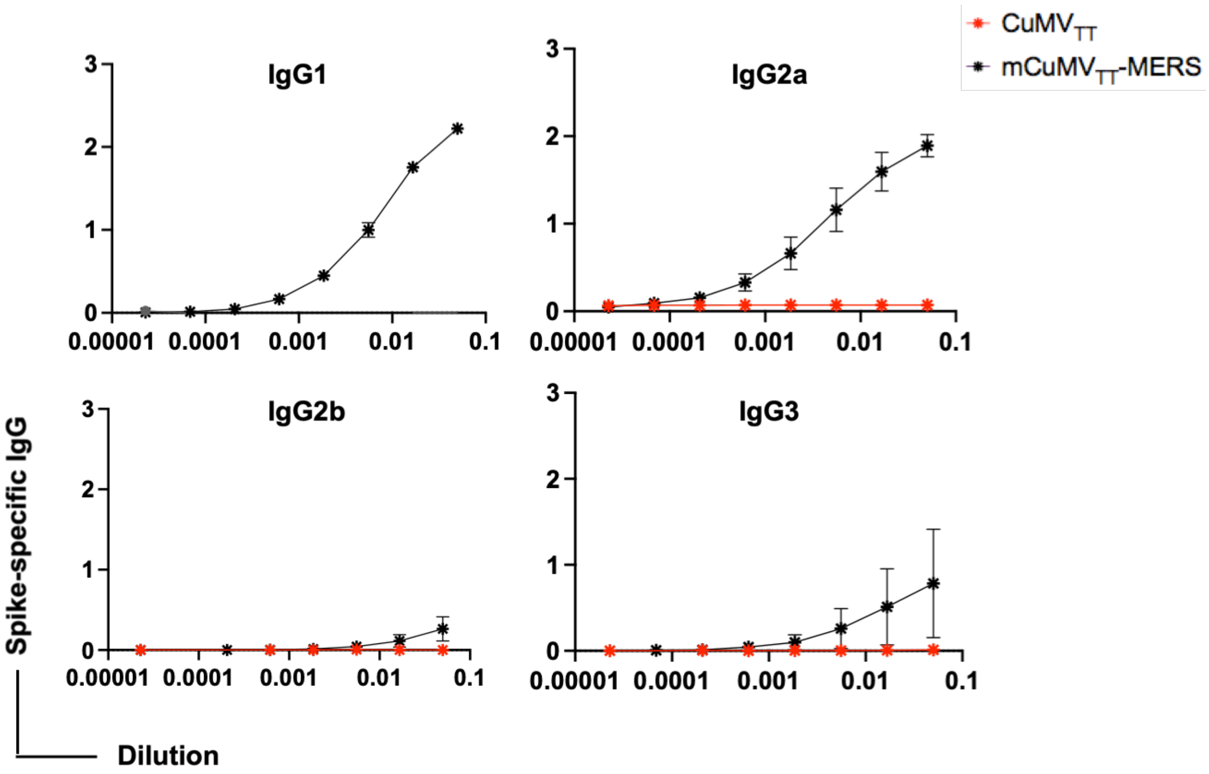

Supplementary Figure 1. Vaccination with mCuMV$_{TT}$-MERS elicits a strong humoral immune response. Spike-specific IgG1, IgG2a, IgG2b and IgG3 titers for the groups vaccinated with CuMV$_{TT}$ control or mCuMV$_{TT}$-MERS measured with OD$_{450}$.
